# Enhanced Diabetes Susceptibility in Community Dwelling Han Elders Carrying the Apolipoprotein E 3/3 Genotype

**DOI:** 10.1371/journal.pone.0151336

**Published:** 2016-03-21

**Authors:** Chun-xia Ban, Li Zhong, Tao Wang, Min-jie Zhu, Jing-hua Wang, Zhen-lian Zhang, Zhe Wang, Ning Su, Yuan-yuan Liu, Yan-chen Shi, Shi-fu Xiao, Xia Li

**Affiliations:** 1 Department of Psychogeriatrics, Shanghai Mental Health Center, Shanghai Jiao Tong University School of Medicine, Shanghai, China; 2 Fujian Provincial Key Laboratory of Neurodegenerative Disease and Aging Research, Institute of Neuroscience, Medical College, Xiamen University, Xiamen, China; University of Catanzaro Magna Graecia, ITALY

## Abstract

Despite Apolipoprotein E (ApoE) being one of the main apolipoproteins in the blood, the association between its genotype and the high cholesterol or blood glucose levels commonly seen in clinical practice is inconclusive. Such research is also lacking in the Han population. The aim of this study was to investigate the association between *APOE* genotype, diabetes, and plasma glucose and lipid levels. We included 243 community-dwelling elderly residents in this study. Participant *APOE* genotypes were assessed and were simultaneously tested for weight, height, blood glucose, triglycerides, cholesterol, and high- and low-density lipoprotein. In addition, gender, age, years of education, cognitive function, and medical history was recorded. Subjects were divided into 3 groups based on *APOE* genotype: *APOE* ε2 group (ε2/ε2 and ε2/ε3), *APOE* ε3 group (ε3/ε3), and *APOE* ε4 group (ε2/ε4, ε3/ε4 and ε4/ε4). Comparisons between groups were conducted for the incidence of diabetes, high blood pressure, and dementia, as well as for differences in body-mass index, fasting plasma glucose, and blood lipids. The *APOE* ε3/ε3 genotype exhibited the highest frequency (70.4%) among the subjects. Participants in the *APOE* ε3 group demonstrated significantly higher levels of fasting plasma glucose than those in the *APOE* ε2 and *APOE* ε4 groups (*P*<0.05). The *APOE* ε3 group had slightly higher abnormal fasting plasma glucose values than did the *APOE* ε2 group (*P* = 0.065). Furthermore, the *APOE*3 genotype was significantly correlated with both fasting plasma glucose level and glucose abnormality (*P*< 0.05) and trended toward statistically significant correlation with diabetes (*P* = 0.082). The correlation between *APOE*2 and low low-density lipoprotein levels also approached statistical significance (*P* = 0.052). Thus, elderly community dwelling residents of Han ethnicity carrying the *APOE* ε3/ε3 genotype might have higher plasma glucose levels and a higher occurrence of diabetes.

## Introduction

With the rapid aging of the global population, the prevalence of chronic diseases such as diabetes and cardiovascular disease is increasing every year [1–3] and has become a worldwide public health problem [2]. In 2010, 284.8 million patients suffered with diabetes worldwide in 2010 and this number is predicted to increase to 438.7 million by 2030 [4], with the burden of disease being especially heavy in developing countries [5]. Additionally, the risk of cardiovascular disease greatly increases with diabetes progression [6], patients with diabetes are more likely to present with dyslipidemia than those without [7], and approximately 75 to 80% of individuals with diabetes die from cardiovascular disease [8]. Although considerable advances have been recently attained in diabetes research, the specific mechanism underlying diabetes has yet to elucidated.

Apolipoprotein E (*APOE*) is a protein that is rich in arginine and originates from very low density lipoprotein (VLDL) in normal individuals [9]. *APOE* is related to proteins such as the LDL receptor and VLDL receptor ligands, and is primarily synthesized in the liver and the brain. *APOE* regulates plasma lipoprotein metabolism by modifying the storage and distribution of cholesterol and lipids, and is closely related to lipid metabolism and atherosclerosis [10, 11]. Many studies have demonstrated a correlation between *APOE* genotypes and coronary heart disease and Alzheimer’s Disease (AD), especially with the *APOE* ε4 genotype; however, the relationship between *APOE* genotypes and diabetes or blood glucose levels has not been confirmed. Although one study reported that no such association was found [12], others have found that *APOE*4 or *APOE*2 are associated with blood glucose level [13–15]. Notably, the correlation between the *APOE* gene and cognitive function is influenced by age [16]; for example cognitive functioning in young people carrying the *APOE* ε4 alleles is better than that of non-carriers [17], but gradually weakens after age 50 [16], and after age 65 *APOE* ε4 becomes a risk factor for AD. Age also influences the association between *APOE* genotypes and lipid metabolism [18]. Therefore, we supposed that the correlation between APOE genotypes and blood glucose might have been difficult to determine because previous researchers did not consider the effect of age.

To address this issue, we based this study on a sample comprising community dwelling participants aged 60 and above of Han descent and determined the correlation between *APOE* genotypes and fasting plasma glucose and blood lipids in this homogenous cohort.

## Materials and Methods

### Study design and participants

**Fig 1:**
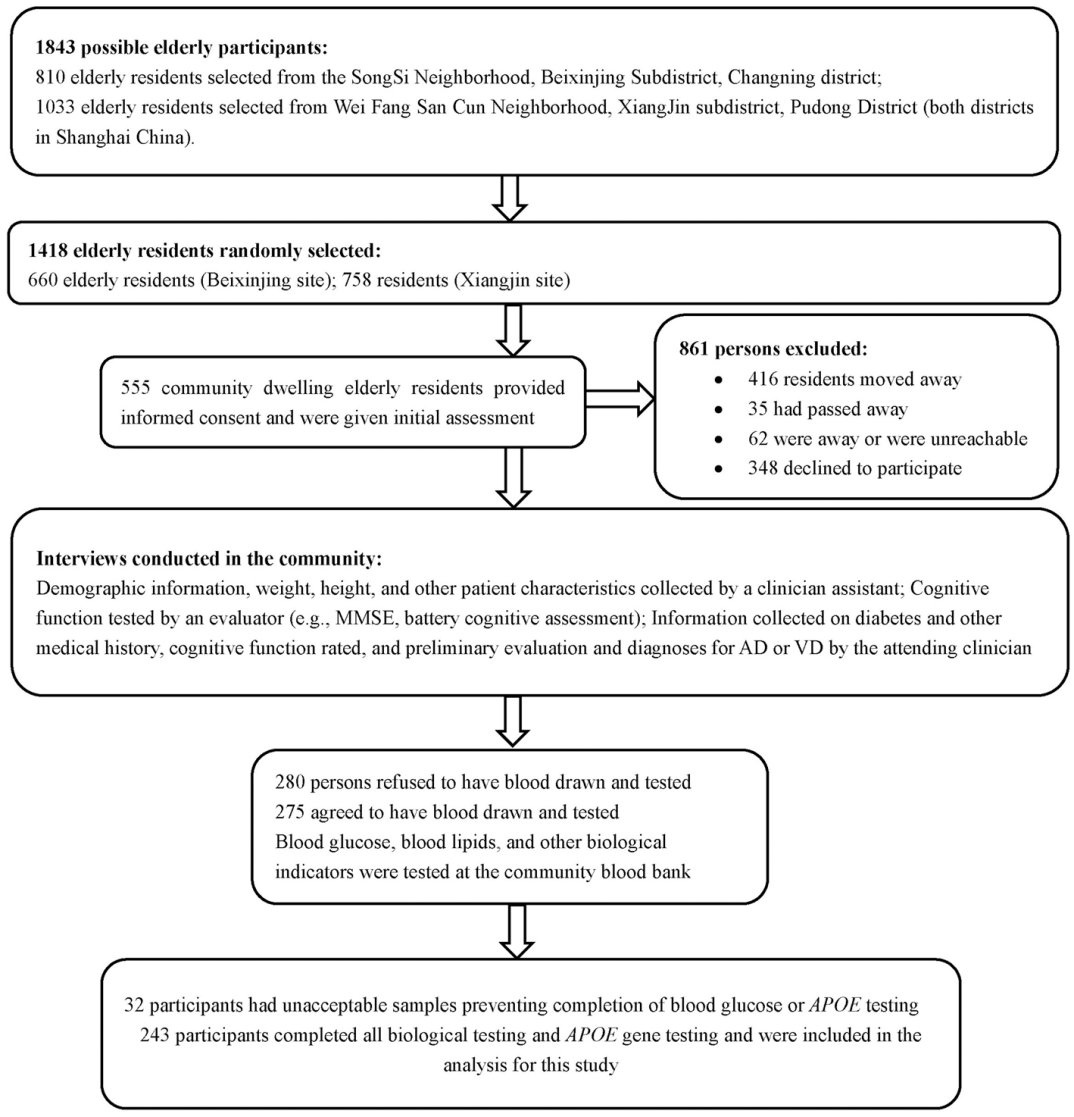
Flowchart of sample selection. MMSE = Mini Mental State Examination; AD = Alzheimer’s Disease; VD = Vascular Dementia; ApoE = Apolipoprotein E.

The study was conducted in two neighborhoods in Shanghai, China (one in the Beixinjing area of the Chang Ning District and the other from the Xiangjing area of the Pudong District) from June to September, 2012. Overall, 810 individuals aged 60 and above resided in the Beixinjing neighborhood whereas 1033 lived in the Xiangjing neighborhood, for a total of 1843 elders. Using a random number chart, 660 residents were selected from the Beixinjing site and 758 from the Xiangjing site for a total of 1418 potential participants. Within this group there were 513 residents who had either moved, passed away, or were unreachable, and 348 residents declined to participate. The remaining 555 residents provided informed consent and were included in the original assessment. Within this group, 280 refused and 275 consented to have blood drawn and tested. However, from the latter group, 32 of the blood samples were not suitable for blood glucose and *APOE* tests. Thus, a total of 243 elderly participants completed blood and *APOE* genetic testing and were included in this study (Fig 1). This study was approved by the ethics committee of the Shanghai Mental Health Center. All participants provided written informed consent.

The 243 final participants were of Han Chinese descent, and consisted of 96 men (39.5%) and 147 women (60.5%) with ages ranging from 60 to 95 years [mean age 71.67 (± 8.331)] and years of education ranging from 0 to 20 [mean 8.36 (± 4. 693)]. Of the 312 individuals that did not enter the study, 135 were men (43.3%) and 177 were women (56.7%), with ages ranging from 60 to 97 years [mean age 72.54 (± 7.983)], and their years of education ranged from 0 to 21 years [mean 9.18 (± 4.721)]. No statistically significant difference (*P* > 0.05) existed between those that entered the study and those that did not enter the study in gender and age; however, the difference in years of education did reach statistical significance (*P* < 0.05).

All general participant information was recorded including gender, age, years of education, height, weight, and body mass index (BMI) was calculated. Included participants received cognitive assessment, including a Chinese version of the Mini Mental State Examination (MMSE) [19], and battery cognitive assessment [20]. Participants with intact activities of daily life and an MMSE score > 24 were considered as having normal cognitive function. AD and vascular dementia (VD) were diagnosed by two senior psychogeriatrists according to the criteria of the Diagnostic and Statistical Manual of Mental Disorders, Fourth Edition (DSM-IV) (1994) and The National Institute of Neurological and Communicative Disorders and the Stroke-Alzheimer's Disease and Related Disorders Association [21]. In addition, the majority of participants with cognitive impairment provided the results of computed tomography or magnetic resonance imaging scans that had previously been performed. Furthermore, comorbidities were recorded through participant report and carefully checked by the senior psychogeriatrists. The diagnosis of diabetes (type 2 diabetes mellitus) was previously administered by endocrinologists according to the criteria from the World Health Organization (WHO 1999) for diabetes diagnosis [22]. High blood pressure, coronary heart disease, hyperlipidemia, and other comorbidities were also recorded as diagnosed by previous clinical specialists.

### Blood glucose and lipid measurements

To determine the levels of blood glucose and lipids, all participants fasted for 12 h and the next morning 4 mL of blood was taken intravenously and stored at room temperature for 30 min before being centrifuged at 1710 × *g* for 15 min to extract the plasma. All participants were tested for plasma glucose, triglycerides, cholesterol, and high- and low-density lipoprotein. Plasma glucose > 6.1 mmol/L was defined as hyperglycemia according to WHO criteria [22].

### *APOE* genotyping by real time polymerase chain reaction (PCR)

We have developed an *APOE* genotyping method based on allele-specific PCR methodology adapted to real time PCR using a TaqMan probe [23]. Briefly, the *APOE* genotyping assay includes three reactions that detect the alleles of *APOE* ε2, ε3, and ε4, respectively. Each PCR reaction mixture (20 μL) contained the following reagents: 1×AccuPower Plus DualStar TM qPCR PreMix (Bioneer, Daejeon, Korea, K-6603), *APOE* primers, and an *APOE* TaqMan probe (FAM labeled), 20% glycerol, and 20 ng genomic DNA. Positive control DNA template (ε2, ε3, and ε4, plasmid DNA) and negative controls (DNA/RNA-free water) were included in each genotyping panel. The PCR amplification protocol was as follows: initial pre-denaturation at 95°C for 5 min, followed by 40 cycles with denaturation at 95°C for 10 s and annealing/extension at 58°C for 1 min. The fluorescence signals were collected during the annealing/extension step, with FAM signal indicating the *APOE* alleles. The amplification was performed using the Applied Biosystems 7500 Fast Real-Time PCR System. All samples were repeated at least twice and the assays were performed by two investigators.

### Data analysis

The Statistical Package for Social Science (SPSS) version 19.0 (SPSS IBM, Inc., Chicago, IL, USA) was used for analysis and processing of the data. *APOE* allele frequencies were calculated using a Hardy-Weinberg equilibrium *χ*^*2*^ test for goodness of fit. Continuous data (age, years of education, fasting plasma glucose, triglycerides, cholesterol, high-density lipoprotein, low-density lipoprotein, and BMI) are showed as the means ± standard deviation, Categorical data (gender, AD diagnosis, comorbidity with diabetes, high blood pressure, coronary heart disease, hyperlipidemia, and fasting plasma glucose >6.1 mmol/L) are expressed as percentages. The *APOE* genotypes were divided into three groups for comparison: the *APOE* ε2 group (ε2/ε2 and ε2/ε3), *APOE* ε3 group (ε3/ε3); and *APOE* ε4 group (ε2/ε4, ε3/ε4, and ε4/ε4). Continuous data was compared among the three group by one-way analysis of variance and multicomparison of fasting plasma glucose was performed using Tamhane’s T2 method, whereas categorical data was compared using a *χ*^*2*^ test. *APOE2*, *APOE3*, and *APOE4* carrier and non-carrier status was converted into a dichotomous variable and then their correlation with plasma glucose, lipid, and BMI was tested using Spearman’s correlation analysis. The significance level was set at *P* < 0.05.

## Results

### *APOE* genotype distribution among community-dwelling elderly

**Fig 2:**
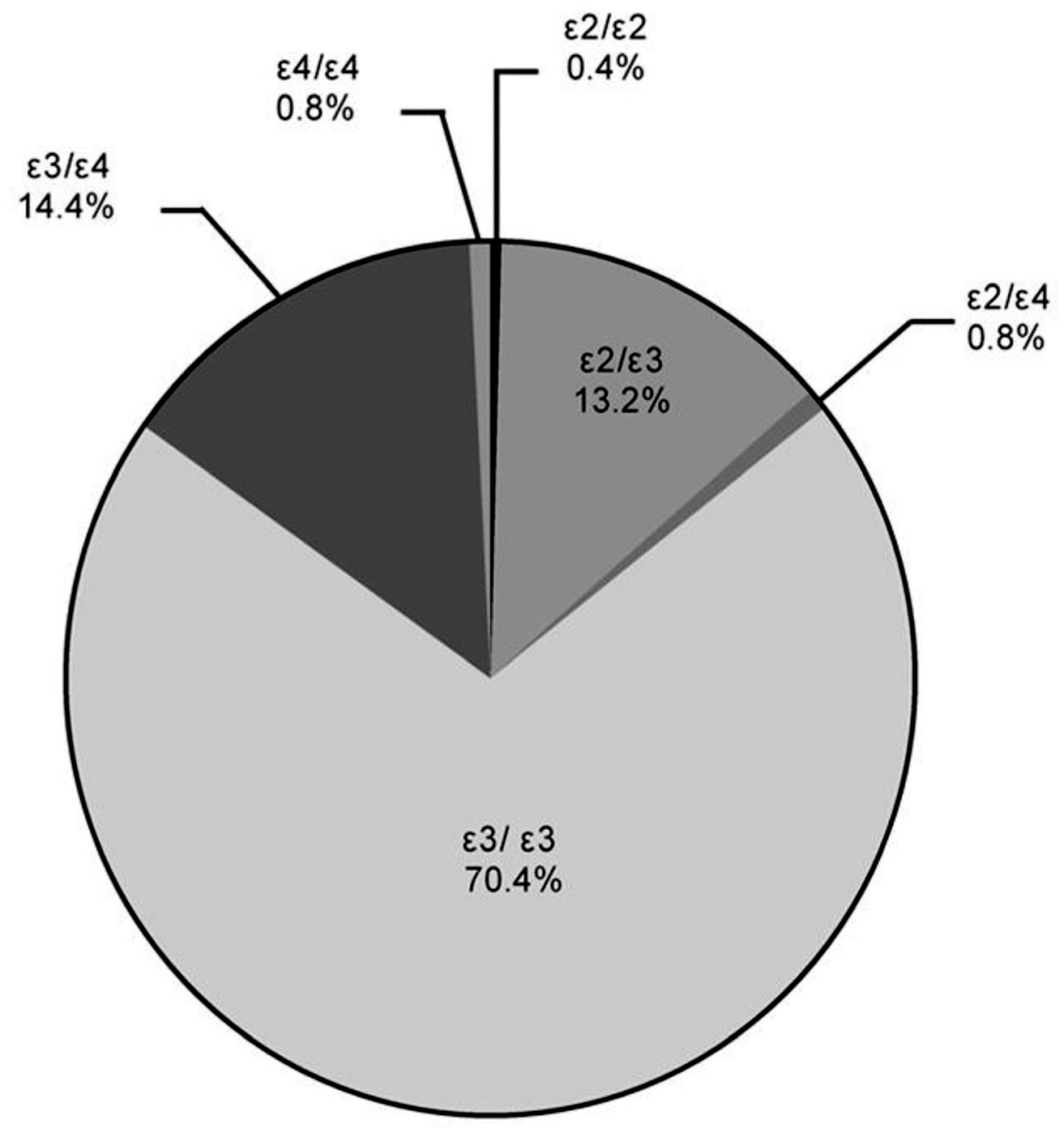
*APOE* genotype distribution in community-dwelling elders.

We identified a total of 6 *APOE* genotypes, among which 1 subject (0.4%) carried ε2/ε2, 32 (13.2%) were ε2/ε3, 2 (0.8%) were ε2/ε4, 171 (70.4%) carried ε3/ε3, 35 (14.4%) were ε3/ε4, and 2 (0.8%) carried ε4/ε4 (Fig 2). A Hardy Weinberg goodness of fit test, *χ*^*2*^ = 0.59, *df* = 3, *P* > 0.05 showed that this cohort exhibited Hardy Weinberg equilibrium. The allele frequencies were as follows: ε2, 36 (7.4%), ε3, 409 (84.2%), and ε4, 41 (8.4%).

### Comparison of *APOE* genotypes with subject characteristics

The difference between fasting plasma glucose in subjects with different *APOE* genotypes was statistically significant (*P* < 0.05), and the fasting glucose level over 6.1 mmol/L almost reached statistical significance (*P* = 0.068). No statistically significant difference was identified between the three groups in gender, age, years of education, presence of AD, high blood pressure, coronary heart disease, diabetes, triglycerides, cholesterol, high-density lipoprotein, low density lipoprotein, or BMI (Table 1).

**Table 1:** Comparison of *APOE* types with patient characteristics.

Item	*APOE* ε2 (n = 33)	*APOE* ε3 (n = 171)	*APOE* ε4 (n = 39)	*x*^*2*^*/F*	*P*-value
Male (%)	11 (33.3%)	69 (40.4%)	16 (41.0%)	0.62	0.735
Mean age (±SD)	71.91 ± 8.10	71.99 ± 8.60	70.03 ± 7.27	0.90	0.407
Mean years of education (±SD)	7.67 ± 4.81	8.20 ± 4.79	9.67 ± 3.98	1.99	0.139
Alzheimer’s disease (%)	1 (3.0%)	12 (7.0%)	4 (10.3%)	1.33	0.528
High blood pressure (%)	20 (60.6%)	89 (52.0%)	22 (56.4%)	0.93	0.627
Coronary heart disease (%)	4 (12.1%)	21 (12.3%)	4 (10.3%)	0.12	1.000
Hyperlipidemia (%)	11 (33.3%)	43 (25.1%)	10 (25.6%)	0.97	0.617
Diabetes (%)	4 (12.1%)	35 (20.5%)	4 (10.3%)	3.09	0.213
Plasma glucose (mmol/L)	5.18 ± 1.10	5.86 ± 2.23	5.17 ± 1.12	3.09	0.047
Plasma glucose >6.1 mmol/L (%)	6 (15.2%)	53 (31.0%)	8 (17.9%)	5.38	0.068
Triglycerides (mmol/L)	1.89 ± 0.98	1.82 ± 1.30	1.90 ± 1.89	0.07	0.932
Cholesterol (mmol/L)	4.89 ± 1.10	4.90 ± 1.15	4.96±0.95	0.06	0.945
High-density lipoprotein (mmol/L)	1.22 ± 0.34	1.18 ± 0.28	1.12±0.23	1.11	0.330
Low-density lipoprotein (mmol/L)	2.72 ± 0.85	2.95 ± 0.94	3.07±0.73	1.44	0.240
BMI (Kg/m^2^) (±SD)	24.58 ± 3.41	24.11 ± 3.36	23.77±3.08	0.53	0.587

*APOE* genotype groups consist of *APOE* ε2 (ε2/ε2 and ε2/ε3); *APOE* ε3 (ε3/ε3); and *APOE* ε4 (ε2/ε4, ε3/ε4, and ε4/ε4). SD = standard deviation; BMI = body mass index.

### Comparison of plasma glucose among different *APOE* carriers

**Fig 3:**
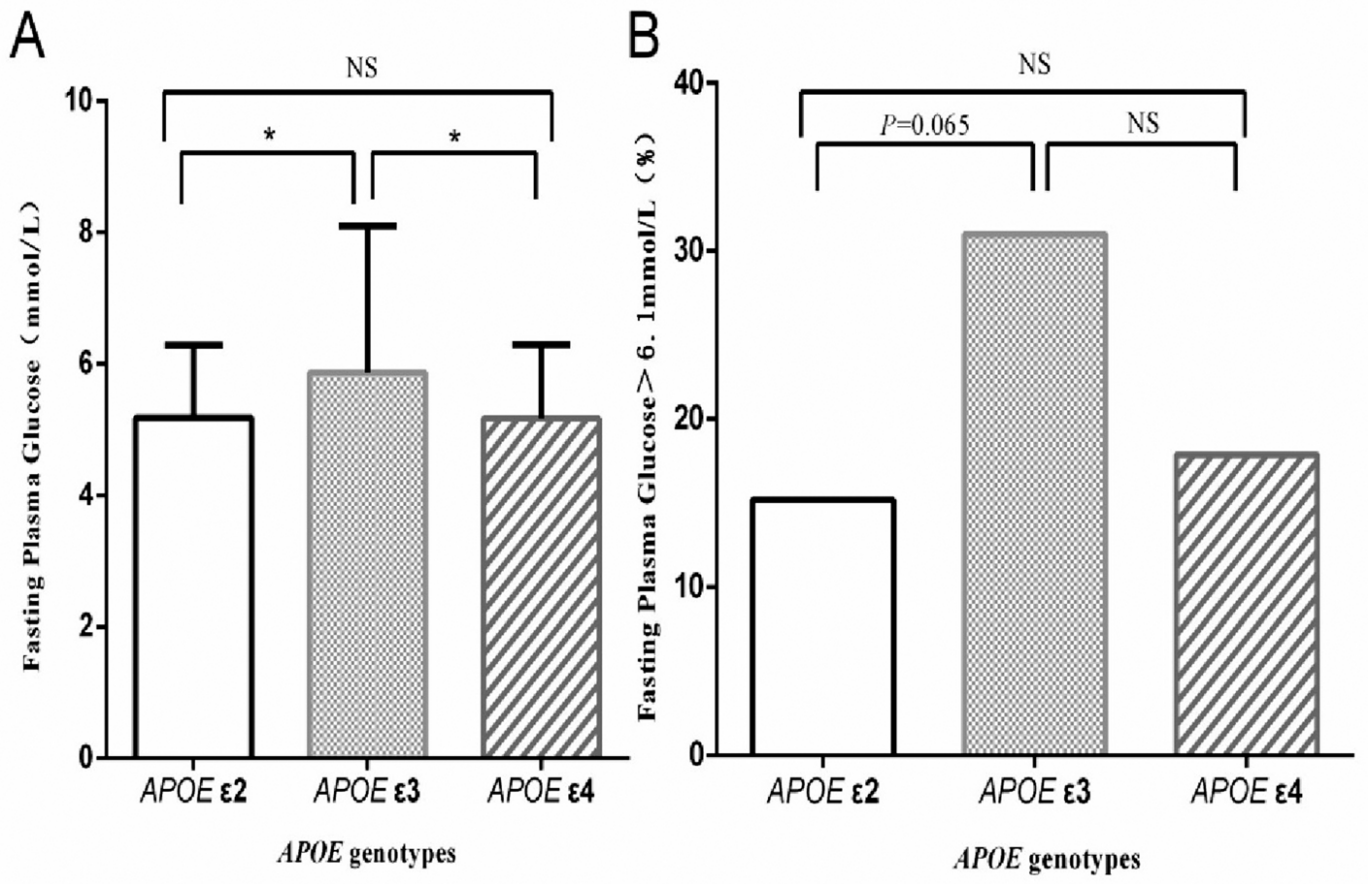
Blood glucose levels of carriers with different *APOE* genotypes. *: *P* > 0.05; NS = no statistical significance.

In comparison with study participants carrying *APOE* ε4 and *APOE* ε2 genotypes, the fasting glucose levels in those with *APOE* ε3 were significantly higher (*P* < 0.05), whereas no significant difference was identified between *APOE* ε2 compared to *APOE* ε4 participants (*P* > 0.05) (Fig 3A). Fasting plasma glucose levels ≤ 6.1 mmol/L are considered normal, whereas higher levels are considered abnormal. Comparisons between genotype groups showed that *APOE* ε3 carriers exhibited abnormal fasting plasma glucose compared to *APOE* ε2 carriers to a degree approaching statistical significance (*P* = 0.065);. differences between the other two groups were not statistically significant (*P* > 0.05) (Fig 3B).

### Correlation between *APOE* alleles and subject characteristics

Carriers of the ε2 allele were defined as *APOE*2 carriers with a variable of 1; non-carriers were given a variable of 0. Those having the ε3/ε3 genotype were defined as *APOE*3 carriers with a variable of 1, those carrying other genotypes were defined as non-*APO*E3 carriers with a variable of 0. Carriers of the ε4 allele were defined as *APOE*4 carriers with a variable of 1, non-carriers were assigned a variable of 0. These three types of carriers and non-carriers were converted into a dichotomous variable and Spearman correlational analysis was performed for plasma glucose, blood lipid, and BMI (Table 2). The presence of *APOE*3 was significantly correlated with abnormal plasma glucose levels (*P* < 0.05) and nearly reached statistical significance (*P* = 0.082) as related to a history of diabetes. In addition, *APOE*2 had a close association with lower levels of low-density lipoprotein (*P* = 0.052). However, *APOE* carrier type was not associated with any other type of lipid level or with a diagnosis of AD (*P* > 0.05).

**Table 2:** Correlation of Elderly Community-dwelling Han Ethnicity *APOE* Carrier Status with Subject Characteristics (*r*).

Item	*APOE*2	*APOE*3	*APOE*4
Fasting plasma glucose			
***r***	−0.077	0.128	−0.101
***P***	0.235	0.047	0.115
Glucose >6.1 mmol/L			
***r***	−0.115	0.148	−0.087
***P***	0.072	0.021	0.177
Presence of diabetes			
***r***	−0.067	0.112	−0.085
***P***	0.296	0.082	0.185
Presence of AD			
***R***	−0.067	0.001	0.056
***P***	0.301	0.984	0.386
Triglycerides			
***r***	0.074	−0.045	−0.014
***P***	0.249	0.488	0.831
Cholesterol			
***r***	−0.039	−0.019	0.035
***P***	0.542	0.768	0.591
High-density lipoprotein			
***r***	0.019	0.025	−0.072
***P***	0.768	0.699	0.263
Low-density lipoprotein			
***r***	−0.125	0.009	0.085
***P***	0.052	0.890	0.189
BMI			
***r***	0.060	- 0.011	−0.037
***P***	0.353	0.863	0.563

AD = Alzheimer’s disease; BMI = body mass index.

## Discussion

There are 6 kinds of common human *APOE* genotypes made up of the 3 *APOE* alleles (ε2, ε3, ε4): ε2/ε2, ε2/ε3, ε3/ε3, ε2/ε4, ε3/ε4, and ε4/ε4. Of these, ε3/ε3 is the most common with a greater than 60% frequency, followed by ε2/ε3 and ε3/ε4; all of these contain the ε3 allele [24], which accordingly exhibits the highest frequency distribution [25]. However, the frequency distributions of the *APOE* alleles differ among various groups and races [26], with the frequency of the ε4 allele in Asians being lower (7.4% in both China and Japan) and in European higher (18.6% in both Finnish and Hungarian) [24]. The two areas of Asia from which the research samples in this study were chosen cover the east and west areas of downtown Shanghai, representing the newer and older districts of the city, respectively. Our research shows that the ε3/ε3 frequency was highest among the *APOE* genotypes detected in this Shanghai-based Han population and the frequency of the ε3 allele was also the highest, similar to that seen in other Asian groups. The *APOE* genotype and allele frequency distributions were found to be in accordance with Hardy-Weinberg equilibrium after examination using the *χ*^2^ test, which indicated the general representativeness of the sample.

Previous research has shown that people with diabetes are more likely to present with dyslipidemia than those without diabetes [7], and that the *APOE* gene is associated with lipid metabolism and heart disease [10, 11, 27]. Therefore, it was speculated that the *APOE* gene might also exhibit a certain relevance to diabetes. However, no agreement has been reached regarding the relationship between *APOE* gene variation and blood glucose level. Research focusing on the relationship between the ε4 allele and plasma glucose [13] has suggested that this allele was related to diabetes with or without the presence of coronary heart disease; however, other studies have found no correlation between the *APOE* gene and blood glucose [12]. Our research first proposed that fasting plasma glucose was higher in the elderly population carrying the *APOE* ε4 allele and that the incidence of diabetes by subjective report in this group was also higher. Notably, our study sample constituted community dwelling elders aged 60 and above; however, previous studies did not include participants in this age group, with an average age of about 50 years old. This difference in age, which has been shown to impact the influence of the *APOE* gene on cognitive function and lipid metabolism [16, 18], might therefore be one of the reasons explaining the difference between our results and those of other studies. In addition, Scuteri et al. [28] conducted long-term follow-up research and discovered that fasting plasma glucose increased with the increase of age for elders carrying *APOE*4+. At baseline, the blood glucose levels were higher in the *APOE*4+ carriers than in the APOE4− carriers, which is not consistent with our results. This inconsistency might be related to race, as the cohort studied by Scuteri consisted primarily of Caucasians, wherein the frequency of the ε4 allele reached 25.5%. In contrast, our subjects were all of Han ethnicity from Asia with an ε4 allele frequency of only 8.4%. On the other hand, one recently published review of Asian populations showed that the ε3 allele was likely related to coronary heart disease [29], indirectly demonstrating that *APOE* ε3/ε3 was a probable risk genotype for glycolipid metabolic disorder in Asian populations. Furthermore, Sapkota et al. [30] investigated an Asian sample and showed that ε2 and ε4-containing genotypes had protective OR of 0.64 in diabetes when compared to the ε3 genotype, which was also consistent with our results. Findings with respect to ε2 have, however, been controversial, as other research has suggested that the ε2 allele is associated with blood glucose and that this allele might instead increase the risk of diabetes [14, 15], which is not consistent with our findings. These differences might also be related to the age or race of the groups used in these studies. Our results support the assertion that *APOE*2 and *APOE*4 are protective factors for diabetes in Asian populations, in concordance with Sapkota’s results; however further research with larger sample sizes is needed to confirm this hypothesis.

Our correlation analyses also showed that ε2 carriers had relatively lower levels of LDLs. APOE is one of the main apolipoproteins in the blood and is related to lipid metabolism abnormalities [31]. Other research has also suggested that ε2 allele could reduce the levels of low density lipoprotein [32] and that ε4 could increase these levels [33]. Notably, lower levels of low density lipoprotein are associated with a lower incidence of coronary heart disease [34]. Although our research showed no difference among the carriers of these three alleles with respect to the incidence of coronary heart disease, it is important to keep in mind that the diagnosis of coronary heart disease in these cases was based on subjective report.

It is known that *APOE* ε4 is a risk factor for the development of AD [35] whereas *APOE* ε2 has a preventive and protective function [36]. Consistent with this, in our study, the results showed that subjects carrying *APOE* ε4 had the highest incidence of AD and that those carrying *APOE* ε2 had the lowest. However, an important note is that the diagnosis of AD was based on clinical interview data obtained from participants by psychiatrists. No further diagnosis was made, so it is therefore possible that other types of dementia such as front temporal lobe dementia and Lewy Body dementia were not fully identified.

This study has several limitations: First, the sample was consisted only of participants of Han ethnicity from the Shanghai area and is not representative of all Han Chinese. Furthermore, the disease history was based on the self-report of community dwelling elderly participants, and therefore included a certain amount of bias. In addition, we lacked other adult samples that could be compared with this elderly sample. These factors need to be improved in future research in order to clarify the impact of *APOE* genotypes on different groups of aged individuals.

## Conclusions

In conclusion, our research is the first study to report that elders of Han ethnicity with the ε3/ε3 genotype are more likely to suffer from diabetes, and that the *APOE* ε2 allele is a possible protective factor for diabetes and blood lipids. This might serve as a new approach to studying the effect of *APOE* on glycolipid metabolism in persons of Han ethnicity and possibly other Asian ethnicities as well.

## Supporting Information

S1 Dataset (SAV)
